# Septin 9 methylated DNA is a sensitive and specific blood test for colorectal cancer

**DOI:** 10.1186/1741-7015-9-133

**Published:** 2011-12-14

**Authors:** Jorja D Warren, Wei Xiong, Ashley M Bunker, Cecily P Vaughn, Larissa V Furtado, William L Roberts, John C Fang, Wade S Samowitz, Karen A Heichman

**Affiliations:** 1 New Technology Group, ARUP Laboratories, Inc., 500 Chipeta Way, Mail Code 209, Salt Lake City, UT 84108-1221, USA; 2 ARUP Institute of Experimental Pathology, ARUP Laboratories, Inc., 500 Chipeta Way, Salt Lake City, UT 84108-1221, USA; 3 University of Utah Department of Pathology, ARUP Laboratories, Inc., 500 Chipeta Way, Salt Lake City, UT 84108-1221, USA; 4 Divison of Gastroenterology, University School of Utah Medicine, 30N 1900 E, Room 4R118, salt Lake City, UT 84132, USA

## Abstract

**Background:**

About half of Americans 50 to 75 years old do not follow recommended colorectal cancer (CRC) screening guidelines, leaving 40 million individuals unscreened. A simple blood test would increase screening compliance, promoting early detection and better patient outcomes. The objective of this study is to demonstrate the performance of an improved sensitivity blood-based Septin 9 (*SEPT9*) methylated DNA test for colorectal cancer. Study variables include clinical stage, tumor location and histologic grade.

**Methods:**

Plasma samples were collected from 50 untreated CRC patients at 3 institutions; 94 control samples were collected at 4 US institutions; samples were collected from 300 colonoscopy patients at 1 US clinic prior to endoscopy. *SEPT9 *methylated DNA concentration was tested in analytical specimens, plasma of known CRC cases, healthy control subjects, and plasma collected from colonoscopy patients.

**Results:**

The improved *SEPT9 *methylated DNA test was more sensitive than previously described methods; the test had an overall sensitivity for CRC of 90% (95% CI, 77.4% to 96.3%) and specificity of 88% (95% CI, 79.6% to 93.7%), detecting CRC in patients of all stages. For early stage cancer (I and II) the test was 87% (95% CI, 71.1% to 95.1%) sensitive. The test identified CRC from all regions, including proximal colon (for example, the cecum) and had a 12% false-positive rate. In a small prospective study, the *SEPT9 *test detected 12% of adenomas with a false-positive rate of 3%.

**Conclusions:**

A sensitive blood-based CRC screening test using the *SEPT9 *biomarker specifically detects a majority of CRCs of all stages and colorectal locations. The test could be offered to individuals of average risk for CRC who are unwilling or unable to undergo colonscopy.

## Background

It has been postulated that a screening test for colorectal cancer (CRC) performed on blood that is collected in the physician's office would encourage more patients to undergo screening, and could significantly decrease CRC mortality. Increased screening would also likely result in cost savings to the healthcare system, since more CRCs would be detected at an earlier stage and newer, more expensive chemotherapies could be avoided [1]. In 2008, Lofton-Day *et al. *[2] described three blood-based molecular biomarkers for CRC that are shed from solid tumors into the bloodstream [3-5]. The same group further developed an assay to detect one of the candidates, Septin 9 (*SEPT9*), which was differentially methylated in CRC tissues [6] and can be sensitively and specifically detected in blood plasma [7,8]. *SEPT9 *DNA methylation was analyzed in several case-control studies, involving more than 3,000 subjects, which demonstrated an overall CRC detection rate of 60 to 70% [7-9]. In 2010, the PRESEPT prospective screening study of the *SEPT9 *biomarker was completed, and the results were presented at the 2010 Digestive Disease Week (DDW) conference [10]. Nearly 8,000 asymptomatic patients from 32 clinical sites in the United States and Germany undergoing routine screening colonoscopy participated in the study [10]. Blood was collected for each subject, and the results of the *SEPT9 *test were compared to colonoscopy with regard to CRC detection [10]. The *SEPT9 *test detected 67% of CRCs and had a false-positive rate of 11% [10], similar to results obtained in the previous case-control studies.

In this report, we describe an improved *SEPT9 *blood-based CRC screening test with a significant increase in sensitivity. Employing recent improvements for duplexing, amplifying and detecting *SEPT9 *methylated DNA, we demonstrate that the new test has dramatically improved performance when compared to the PRESEPT study method. In a case-control study, the new *SEPT9 *test is demonstrated to specifically identify CRCs from blood plasma with sensitivity similar to colonoscopy, exceeding the rates published for stool-based tests and previously described *SEPT9 *blood tests. The ability of the test to detect cancers originating from all large intestine locations is presented.

## Methods

### Human Plasma Samples

The clinical performance of the *SEPT9 *methylated DNA assay was measured using blinded plasma specimens collected from CRC patients and colonoscopy-verified control subjects. Specimens were collected from 50 untreated CRC patients prior to surgery at one US and two Russian institutions between July 2008 and March 2009. The average age of the cancer patients was 62 (range: 42 to 85) years. Control specimens were collected from 94 CRC-free subjects at four institutions in the US within one year of having a negative colonoscopy; collections occurred between July 2008 and June 2010. Control subjects had an average age of 58 (range: 40 to 86) years. A separate set of controls involving 98 younger subjects between the ages of 18 to 49 was collected at ARUP Laboratories between January and April 2011. The average age in this group was 32.

For the small prospective study, blood specimens were collected from 306 patients undergoing colonoscopy at a single community clinic in the US from March to June 2011; 300 of the subjects were evaluable. The average age of the cohort was 56 (range: 22 to 84) years; 195 of these were 50 to 75 (average 59) years of age, were asymptomatic, and underwent a routine screening colonoscopy.

Written informed consent was obtained from all study participants, adhering to local ethics guidelines.

### Laboratory Methods

#### Analytical performance of the blood-based *SEPT9 *assay

Analytical performance of the assay was determined using CpGenome wholly methylated human genomic DNA (Chemicon/Millipore, Billerica, Massachusetts) added to pooled normal human plasma (Innovative Research, Novi, Michigan). The limit of detection of *SEPT9 *methylated DNA at the specimen level was 6.25 pg/ml (at least one out of the three reactions had *SEPT9 *detected 100% of the time). The limit of detection at the PCR replicate level was 50 pg/ml (all three out of three reactions had *SEPT9 *detected 100% of the time). For the comparison study with the PRESEPT Epi proColon PCR method, concentrations ranging from 6.25 to 100 pg/ml of wholly methylated human genomic DNA were used. DNA was extracted from multiple aliquots of each concentration, treated with bisulfite, and purified. Resultant DNA samples from each concentration were pooled, so that the same DNA substrate was used in the PCR method comparisons.

#### DNA Preparation and Bisulfite Conversion from Plasma Specimens

For each subject, 10 ml of blood was collected in an EDTA (ethylenediaminetetraacetic acid) vacutainer tube. Each tube was centrifuged for 12 minutes at 1350 × g ± 150 × g at room temperature. Plasma was transferred without disturbing the buffy coat to a clean 15 ml conical tube. The sample was centrifuged a second time for 12 minutes at 1350 × g ± 150 × g. Plasma was transferred without disturbing the pellet to a 4 ml tube and stored at -70°C. Total genomic DNA was extracted from 4 ml of plasma using a nucleic acid extraction kit from Chemagen (Chemagic NA Extraction kit catalog number 1045 distributed by PerkinElmer, Waltham, Massachusetts) following the product insert protocol. Sample DNA was treated with bisulfite conversion reagents prepared according to the protocol from deVos *et al. *[8]. All bisulfite reagents were purchased from Sigma-Aldrich (St. Louis, Missouri). After bisulfite conversion, samples were purified using a bisulfite purification kit from Chemagen (Chemagic Bisulfite Purification Kit number 1036) following the product insert protocol. DNA was eluted in 55 μL of elution buffer. If not used immediately, eluted DNA was stored at -20°C for up to one week.

#### Real-Time PCR

PCR amplification was performed in triplicate for each sample using a modified version of the protocol from deVos *et al. *[8]. Septin 9 (*SEPT9*) and beta-actin (*ACTB*) control reactions were performed in the same reaction. All primers and probes were synthesized by Integrated DNA Technologies (Coralville, Iowa). Qiagen (Germantown, Maryland) 2X QuantiTect Multiplex Kit No ROX was used. The total volume of the PCR was 25 μL using 10 μL DNA and 12.5 μL 2X QuantiTect Kit. Sequences and final concentrations were as follows: *SEPT9*-FWD AAATAATCCCATCCAACTA (1.5 μM), *SEPT9*-REV GATT-dSp-GTTGTTTATTAGTTATTATGT (1.5 μM), *SEPT9*-Blocker GTTATTATGTTGGATTTTGTGGTTAATGTGTAG-SpC3 (1.0 μM), *SEPT9*-Probe FAM-TTAACCGCGAAATCCGAC-BHQ_1 (0.075 μM), *ACTB*-FWD GTGATGGAGGAGGTTTAGTAAGTT (0.2 μM), *ACTB*-REV CCAATAAAACCTACTCCTCCCTTAA (0.2 μM), *ACTB*-probe TEX615-ACCACCACCCAACACACAATAACAAACACA-IAbRQSp (0.075 μM). Real-time PCR was performed on the LC480 thermal cycler (Roche Applied Science, Indianapolis, Indiana) using the following cycling conditions: activation at 95°C for 30 minutes, 50 cycles of 95°C for 10 seconds, 56°C for 30 seconds, and final cooling to 40°C for 30 seconds. Heating rates were 4.4°C/second and cooling rates, 2.2°C/second. Data were acquired at the end of each 56°C step. Samples were analyzed using the AbsQuant/2^nd^DerivativeMax function of the LC480 software. For the comparison study, the PCR method was performed as described in the Epi proColon Instructions For Use pamphlet (Epigenomics AG, Berlin, Germany). Analysis was done using the AbsQuant/Fit points function of the LC480 software following the Epi proColon real-time PCR protocol.

### PCR Data Analysis

In order to maximize sensitivity, *SEPT9 *was called positive if at least one of the triplicate reactions had detectable *SEPT9*. For plasma specimens that contain very low levels of DNA, *SEPT9 *was 'detected' if the quantification cycle ('crossing point', CP) was less than 45 cycles, the highest value reliably measured by the LC480 AbsQuant/2^nd^DerivativeMax analysis function. Plasma specimens were 'not detected' if the *SEPT9 *CP was not measurable or was ≥ 45.0 cycles and the *ACTB *CP was ≥ 36.0 cycles. If *ACTB *was not detected, eluted DNA specimens were diluted 1:10 in water and re-run; for these studies, a CP of 39.0 cycles for *ACTB *was the maximum value accepted in order to confirm a *SEPT9 *negative result.

### Statistical Analysis

In the case control study, the sensitivity and specificity of the *SEPT9 *test for detecting CRC were calculated as follows:

Sensitivity = true positives∕total cancers

Specificity = false positives∕total controls

95% confidence intervals were calculated according to the efficient-score method (corrected for continuity) [11,12]. Negative and positive predictive values were calculated as follows:

$$\text{Negative predictive value }\left(\text{NPV}\right)\;=\text{ true negatives}∕\text{true negatives }+\text{ false negatives}$$

$$\text{Positive predictive value }\left(\text{PPV}\right)\;=\text{ true positives}∕\text{true positives }+\text{ false positives}$$

For these NPV and PPV calculations, the prevalence of CRC in the screening population was assumed to be 1 in 200 based on the work of Lieberman [13].

## Results

### Analytical performance of the improved blood-based *SEPT9 *test

**Figure 1:**
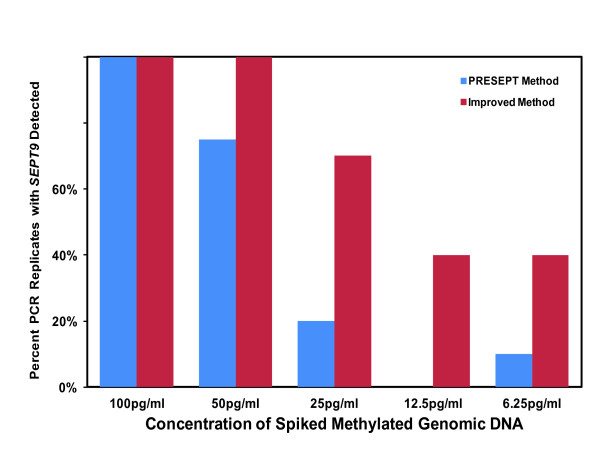
**Comparison of the Improved *SEPT9 *PCR Detection Protocol with the PRESEPT Method using Analytical Specimens**. Methylated *SEPT9 *DNA was measured in pooled normal human plasma spiked with various concentrations of wholly methylated human DNA. Multiple samples of each DNA concentration were prepared and pooled, allowing for the direct comparison of the PRESEPT and improved PCR detection methods using identical DNA substrates. PCR, polymerase chain reaction.

The *SEPT9 *test presented in this publication was specifically developed to improve upon the method described by deVos [8] that was used in the PRESEPT screening study [10]. Each element of the deVos PCR detection protocol was specifically optimized, followed by the development of a robust duplex reaction whereby both *SEPT9 *and *ACTB *(internal control) were measured in the same reaction using different fluorescent tags, similar to the PRESEPT method. Both methods use essentially the same reagents and can be completed in roughly 24 hours, with approximately four hours of hands-on time. The critical alterations compared to the deVos and PRESEPT protocols are as follows: 1) *SEPT9 *PCR primer concentrations were increased; 2) *ACTB *PCR primer concentrations were decreased; and 3) a different fluorescent label was used for the *ACTB *probe. Figure 1 demonstrates the sensitivity of the improved protocol compared to the PRESEPT method using pooled human plasma spiked with various concentrations of methylated human DNA. The increased sensitivity of our method is exemplified by the 6.25 and 12.5 pg/ml dilutions, whereby the improved protocol detected *SEPT9 *in an average of 40% of the replicates, while the PRESEPT method was only capable of detecting *SEPT9 *an average of 5% of the time. Further, the detection of the *SEPT9 *signal occurred several cycles earlier in the improved PCR method compared to the PRESEPT protocol, demonstrating an increased sensitivity of at least ten-fold (Additional file 1, Supplementary Table 1).

### Clinical performance of the blood-based *SEPT9 *test

**Figure 2:**
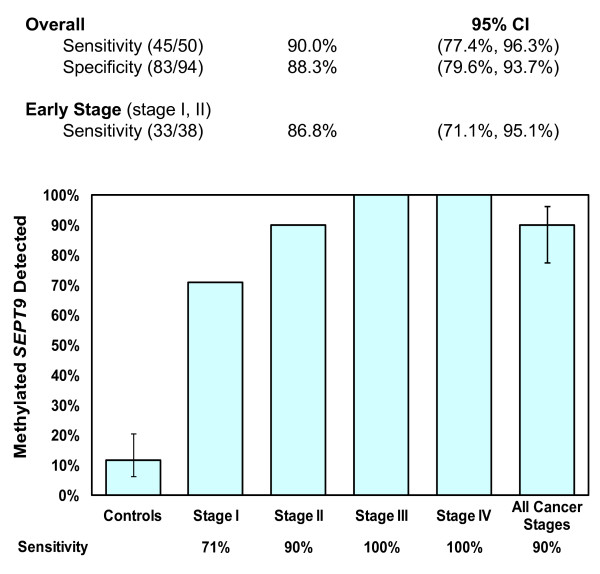
**Sensitivity of the *SEPT9 *Blood-Based Test in Clinical Case-Control Study of 144 Subjects**. Methylated *SEPT9 *DNA was measured in plasma specimens donated by CRC patients and colonoscopy-confirmed control subjects. The percent of specimens with detectable methylated *SEPT9 *DNA is illustrated by the solid bars. The test has been maximized for sensitivity by only requiring one out of three of the PCR replicates to have methylated *SEPT9 *DNA detected. The specificity of the assay is 88% under these parameters. CRC, colorectal cancer; PCR polymerase chain reaction.

**Figure 3:**
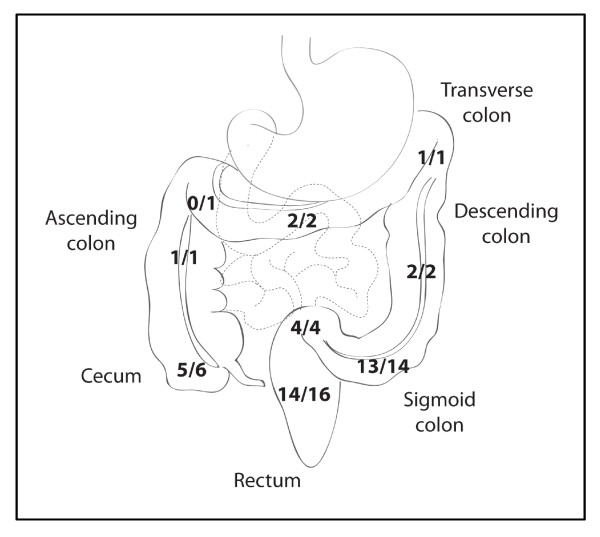
**Location of Tumors Detected by *SEPT9 *Blood-Based Test**. The diagram illustrates the locations of the primary tumors that were detected using the blood-based methylated *SEPT9 *DNA test. Note that CRCs were identified throughout the large intestine, including proximal regions such as the cecum. Three of the fifty blood specimens did not have tumor locations recorded, therefore these specimens are not represented by this figure. CRC, colorectal cancer.

In a case-control study using specimens blinded to the operator, the optimized *SEPT9 *test was able to identify 45 out of 50 cancers from the plasma of CRC patients, with an overall sensitivity of 90% (95% confidence intervals 77.4% to 96.3%). Three-quarters of the samples were contributed by patients with early stage disease (stages I and II), approximating the stage of disease typically detected during routine screening. The new *SEPT9 *test was able to detect 33 out of the 38 of these early cancers for a sensitivity of 87%, and detected all late stage cancers (stages III and IV). Additional file 2, Supplementary Table 2 lists the subjects that participated in the study, including demographic and clinical information, together with *SEPT9 *and *ACTB *crossing points. *SEPT9 *was 'detected' if the crossing point of at least one out of three PCR replicates for each specimen had a value of less than 45 cycles. Figure 2 is a diagram illustrating the overall performance of the *SEPT9 *test, with each cancer stage shown individually. The *SEPT9 *test detected CRCs arising from all regions of the colon and rectum, including proximal tumors arising from the cecum and ascending colon (Additional file 2, Supplementary Table 2). Figure 3 illustrates the cancer detection frequency for each of the regions. The ten percent of tumors that were not detected by the assay were from a variety of regions of the lower GI tract. *SEPT9 *methylation was detected in 11 out of 94 of the control specimens collected from CRC-free colonoscopy-verified individuals age 40 and older (Additional file 3, Supplementary Table 3). The test had an overall specificity of 88%, consistent with previous reports [2,7-9]. This false-positive rate of 12% was relatively stable across different age cohorts from 50 to 75, with only a slight increase to 12.5% in the age 69 to 75 subset. In a separate study, *SEPT9 *methylation was tested in 98 healthy younger control subjects with no personal or family history of CRC between the ages of 18 to 49; in this group, *SEPT9 *methylated DNA was detected in 6% of the subjects (data not shown).

In order to gauge whether the improved *SEPT9 *test might be useful for detection of adenomas, blood was collected from 306 colonoscopy patients at a community clinic, none of whom were shown by colonoscopy to have colorectal cancer. In the entire set of 300 evaluable subjects (ages 24 to 83 years, average 56), only 12% of the 104 subjects with adenomas were detected using the *SEPT9 *test (Table 1). The test was again shown to be specific, with an overall false-positive rate of 3%. Similarly, in the true screening population, those asymptomatic individuals age 50 to 75 years (average 58), only 10% of individuals with adenomas were detected using the blood test. Although the adenoma detection rate was very low, more than half of the subjects in this study with detectable *SEPT9 *were found by colonoscopy to possess an adenoma or other polyp. The most common cause of a false-positive result was diverticulosis, which accounted for nearly half of the 3% false-positive rate.

**Table 1:** *SEPT9 *detection in specimens collected prospectively from colonoscopy patients

	All Evaluable Subjects (age 24-86 years)		Asymptomatic Subjects (age 50-75 years)	
	**Total**	***SEPT9 *+**	**Total**	***SEPT9 *+**

Total subjects	300	21	195	15
Colorectal cancer	0	0	0	0
Other cancer (carcinoid tumor)	1	0	0	0
Adenoma	104	12	78	8
Adenoma ≤ 10 mm	93	11	69	7
Adenoma > 10 mm	11	1	9	1
Hyperplastic/other polyp	38	1	27	1
Diverticulosis	43	4	27	3
Hemorrhoids	29	1	12	0
Crohn's disease/colitis	7	0	3	0
Other colonic findings	21	0	9	0
Unsatisfactory prep/aborted	11	0	9	0
Normal	53	3	34	3

### Discussion

In several clinical studies, which together include over 10,000 subjects, *SEPT9 *has consistently demonstrated utility for detecting CRC in the blood, with previous publications citing a rate of 60% to 70% [2,7,8,10]. In the PRESEPT prospective study of nearly 8,000 asymptomatic individuals undergoing routine CRC screening, the CRC detection rate was 67% with a specificity of 89%, similar to results obtained in case-control studies [10]. Our publication describes an improved *SEPT9 *blood test with enhanced sensitivity, proven by direct comparison with the PRESEPT method using identical analytical specimens. In a case-control study of 144 blinded specimens, the improved *SEPT9 *blood test detected cancers of all stages and colorectal locations, including 87% of early stage cases (stages I and II). The new test exhibited an overall CRC detection rate of 90% at 88% specificity, contrasting historical studies of *SEPT9*.

The *SEPT9 *test is performed as a duplexed PCR, with each reaction run in triplicate to maximize the amount of DNA specimen analyzed. As was originally described in previous *SEPT9 *methods, the improved test is currently configured to favor sensitivity over specificity, whereby *SEPT9 *must be detectable in only one out of three PCR replicates in order to call a positive test. Assuming a CRC prevalence of 0.5% in the screening population of individuals age 50 or older [13], and similar test performance in the screening setting as the case-control study presented here, the improved test would have a negative predictive value of 99.94% (Table 2). A more stringent variation of the test could be used to maximize specificity to 100%, resulting in a positive predictive value of 100%, however the sensitivity would be reduced to 70%. While a 70% detection rate with 100% specificity might out-perform many of the other laboratory-based screening tests for detecting CRC, it is our belief that a maximally sensitive method that would detect a majority of cancers in their early stages would provide a better screening option for the millions of otherwise unscreened individuals.

**Table 2:** Summary statistics for the *SEPT9 *test in colorectal cancer detection

	Most Sensitive	Moderate Sensitivity, Moderate Specificity	Most Specific
Positive PCR replicates	1 out of 3	2 out of 3	3 out of 3
Sensitivity	90%	76%	70%
Specificity	88%	99%	100%
Positive predictive value	3.61%	26.30%	100%
Negative predictive value	99.94%	99.88%	99.85%

Although tissue studies showed that adenomas have elevated levels of methylated *SEPT9 *DNA comparable to CRCs (data not shown), the adenoma detection rate in the plasma was a modest 10% to 12%, consistent with previous studies of the *SEPT9 *biomarker [7,10] and similar to that reported for a standard guaiac fecal occult blood test (FOBT) [13]. Note that these early FOBT tests, which have been reported to detect lower percentages of colorectal cancers [13], were shown in several large prospective screening studies to provide a survival benefit to those who underwent screening when compared to those who did not [14-17]. There did not appear to be any specific types or size of adenomas that were more amenable to *SEPT9 *detection, although a more extensive study with larger numbers of specimens will be required. These results suggest that while the new method is very useful for detecting a majority of CRCs of all stages and locations from the plasma, a blood-based test for *SEPT9 *alone will not be sufficient to detect mucosal precancerous lesions.

Methylated *SEPT9 *may normally play a role in embryonic development in humans. In evaluating *SEPT9 *methylation in normal healthy young control subjects under the age of 50 years, four women demonstrated high concentrations of methylated *SEPT9 *DNA (Warren *et al.*, unpublished data). These women were subsequently found to be pregnant. Additional studies with 20 pregnant women showed that 100% of these subjects had very high concentrations of methylated *SEPT9 *in their plasma. Like other well-known cancer biomarkers such as alpha-fetoprotein (AFP), carcinoembryonic antigen (CEA) and CA-125, *SEPT9 *is implicated in both embryogenesis and oncogenesis. Future studies are planned to determine whether the sensitive *SEPT9 *blood test might be useful for therapeutic monitoring and early detection of relapse, such as CEA and CA-125.

## Conclusions

Although it is clear that CRC screening reduces mortality by detecting the disease in its earliest stages when it is most effectively treated [14-28], only one half of Americans age 50 and older currently undergo any kind of screening [29,30]. Patient compliance appears to be a major hurdle [31]. Even those individuals who otherwise adhere to screening recommendations for other cancers, such as those who routinely undergo mammography, do not faithfully follow colorectal screening guidelines [31]. Physician recommendation plays a significant role in whether individuals are screened [32], however patient preference appears to strongly determine what method, if any, is ultimately used [33]. Reasons for not complying with colonoscopy referral include the time-consuming nature of the procedure and concern about invasiveness [33]. Alternative methods for CRC screening such as fecal testing have declined in recent years [32]. In addition to the challenges of patient compliance with stool testing, such as the requirement for multiple samples and the handling of specimens, the performance of these tests is quite variable, with cancer detection rates ranging from 30% to 85% (13). Newer stool based tests such as the immunochemical FOBT (FIT), have demonstrated sensitivity for adenoma detection [1]. While the *SEPT9 *methylated DNA test may perform comparably to colonoscopy in detecting CRCs, it lacks the advantage of being potentially curative, and does not perform well for adenoma detection. Nonetheless, we believe that a blood-based CRC test, whereby specimens are collected in the primary care setting every two or three years, will attract a significant fraction of those individuals who are otherwise non-compliant with recommended screening guidelines. Studies are underway to gain a better understanding as to whether a blood-based test will encourage individuals in the average risk screening population to undergo testing of this type.

## Abbreviations

*ACTB*: beta-actin gene; AFP: alpha-fetoprotein; avg: average; C: centrigrade; CEA: carcinoembryonic antigen; CA-125: carbohydrate antigen 125; CI: confidence interval; CP: crossing point; CRC: colorectal cancer; DDW: Digestive Disease Week; DNA: deoxyribonucleic acid; FIT: fecal immunochemical test; FOBT: fecal occult test; g: gravity; GI: gastrointestinal: pg: picogram; μl microliter; μM: micromolar; ml: milliter; NPV: negative predictive value; PCR: polymerase chain reaction; PPV: positive predictive value; *SEPT9*: septin 9 gene; US: United States.

## Competing interests

This study was funded exclusively by ARUP Laboratories, Inc., a non-profit national reference laboratory, which is a wholly owned enterprise of the University of Utah and its Department of Pathology. The study was designed and performed by ARUP employees and members of the University of Utah School of Medicine. None of the authors have any competing interests.

## Authors' contributions

JDW and WX developed the improved *SEPT9 *protocol. JDW performed all *SEPT9 *assays. CPV prepared tissue specimens for DNA extraction. LVF and WSS analyzed colon tissue specimens. AMB handled blood specimen collection, plasma sample preparation, and patient consent. KAH, WLR, WSS and JDW contributed to the clinical study design and experimental design. JCF contributed to the colonoscopy study design and specimen collection. KAH and JDW drafted the manuscript. All authors read, provided critical input, and approved the manuscript.

## Pre-publication history

The pre-publication history for this paper can be accessed here:

http://www.biomedcentral.com/1741-7015/9/133/prepub

## Supplementary Material

Additional file 1 **Supplementary Table 1**. Comparison of improved *SEPT9 *detection protocol with PRESEPT method using analytical specimens. Click here for file

Additional file 2 **Supplementary Table 2**. Measurement of *SEPT9 *methylated DNA in plasma of colorectal cancer patients. Click here for file

Additional file 3 **Supplementary Table 3**. Control Plasma Specimens. Click here for file
