# Supplementary material for: Septin 9 methylated DNA is a sensitive and specific blood test for colorectal cancer
Source: BMC Med. 2011 Dec 14;9:133. doi: 10.1186/1741-7015-9-133 (PMC3271041; doi:10.1186/1741-7015-9-133)
Supplement: Additional file 1 — Supplementary Table 1. Comparison of improved SEPT9 detection protocol with PRESEPT method using analytical specimens. [file 1741-7015-9-133-S1.DOC]

**Supplementary Table 1. Comparison of Improved *SEPT9* Detection Protocol with PRESEPT Method using Analytical Specimens**

| **Concentration** | **PRESEPT**  ***SEPT9* detected** | **PRESEPT**  ***SEPT9* Avg CP** | **New Method**  ***SEPT9* detected** | **New Method**  ***SEPT9* Avg CP** |
| --- | --- | --- | --- | --- |
| **6.25 pg/ml** | 2 out of 20 | 49.7 | 8 out of 20 | 39.4 |
| **12.5 pg/ml** | 0 out of 20 | ND | 8 out of 20 | 38.8 |
| **25 pg/ml** | 4 out of 20 | 44.4 | 14 out of 20 | 37.9 |
| **50 pg/ml** | 15 out of 20 | 47.4 | 20 out of 20 | 37.1 |
| **100 pg/ml** | 12 out of 12 | 43.9 | 12 out of 12 | 36.4 |
| **Positive workflow control** | 12 out of 12 | 40.8 | 12 out of 12 | 35.3 |
| **Negative workflow control** | 0 out of 12 | ND | 0 out of 12 | ND |

CP – crossing point

ND – not detected
