# Supplementary material for: Septin 9 methylated DNA is a sensitive and specific blood test for colorectal cancer
Source: BMC Med. 2011 Dec 14;9:133. doi: 10.1186/1741-7015-9-133 (PMC3271041; doi:10.1186/1741-7015-9-133)
Supplement: Additional file 2 — Supplementary Table 2. Measurement of SEPT9 methylated DNA in plasma of colorectal cancer patients. [file 1741-7015-9-133-S2.DOC]

**Supplementary Table 2. Measurement of *SEPT9* methylated DNA in plasma of colorectal cancer patients**

| **ID** | **Stage** | **Location of tumor** | **Age** | **Sex** | ***SEPT9* CP 1** | ***SEPT9* CP 2** | ***SEPT9* CP 3** | ***ACTB*  CP 1** | ***ACTB* CP 2** | ***ACTB* CP 3** | ***SEPT9* Detection** |
| --- | --- | --- | --- | --- | --- | --- | --- | --- | --- | --- | --- |
| 1 | I | Rectum | 49 | F | ND | 38.84 | ND | 30.50 | 30.29 | 30.12 | detected |
| 2 | I | Ascending | 71 | F | 38.07 | 37.92 | 36.78 | 31.91 | 31.81 | 32.12 | detected |
| 3 | I | Rectum | 70 | M | ND | ND | ND | 28.41 | 28.34 | 28.16 | ND |
| 4 | I | Rectum | 57 | F | 37.62 | 36.68 | 37.66 | 31.23 | 31.18 | 31.19 | detected |
| 5 | I | Rectum | 55 | M | 26.05 | 26.07 | 26.12 | 25.94 | 25.85 | 25.90 | detected |
| 6 | I | Rectum | 55 | M | 26.52 | 26.60 | 26.53 | 26.3 | 26.48 | 26.28 | detected |
| 7 | I | Rectum | 64 | F | ND | ND | ND | 31.52 | 31.64 | 31.73 | ND |
| 8 | II | Sigmoid | 78 | F | 34.49 | 34.59 | 34.1 | 30.78 | 30.83 | 30.72 | detected |
| 9 | II | Sigmoid | 78 | F | 34.61 | 34.59 | 34.45 | 31.70 | 31.68 | 31.70 | detected |
| 10 | II | Cecum | 68 | M | ND | 38.33 | ND | 31.27 | 31.48 | 31.55 | detected |
| 11 | II | Sigmoid | 45 | M | 38.56 | 37.48 | ND | 31.81 | 32.04 | 31.89 | detected |
| 12 | II | Transverse | 80 | M | 35.59 | 34.60 | 35.36 | 31.20 | 31.09 | 31.23 | detected |
| 13 | II | Rectum | 60 | F | 36.86 | 36.92 | 37.21 | 26.04 | 26.18 | 26.11 | detected |
| 14 | II | Rectum | 54 | M | 38.57 | ND | ND | 30.69 | 30.82 | 30.83 | detected |
| 15 | II | Sigmoid | 57 | M | 38.55 | 36.07 | 37.94 | 31.93 | 31.85 | 32.17 | detected |
| 16 | II | Sigmoid | 75 | F | 36.57 | 38.17 | 37.56 | 31.97 | 31.98 | 31.99 | detected |
| 17 | II | Sigmoid | 48 | M | 35.48 | 34.99 | 35.77 | 30.46 | 30.44 | 30.32 | detected |
| 18 | II | Rectum | 47 | M | 34.85 | 36.21 | 36.08 | 28.73 | 28.65 | 28.82 | detected |
| 19 | II | Sigmoid | 53 | F | 33.18 | 34.00 | 33.72 | 31.17 | 31.29 | 31.16 | detected |
| 20 | II | Sigmoid | 50 | M | 36.29 | 35.26 | 37.00 | 30.95 | 31.08 | 31.05 | detected |
| 21 | II | Descending | 85 | M | ND | 37.73 | 38.05 | 32.08 | 32.11 | 32.08 | detected |
| 22 | II A | Sigmoid | 70 | M | 38.51 | 36.05 | 36.55 | 31.82 | 31.94 | 31.78 | detected |
| 23 | II A | Descending | 60 | F | 36.18 | 36.31 | 36.75 | 30.86 | 30.97 | 30.96 | detected |
| 24 | II A | Sigmoid | 53 | F | ND | ND | 37.97 | 31.94 | 32.08 | 32.02 | detected |
| 25 | II A | Cecum | 69 | F | ND | 37.82 | ND | 31.12 | 31.16 | 31.1 | detected |
| 26 | II A | Rectum | 59 | M | 29.93 | 29.97 | 30.13 | 29.6 | 29.64 | 29.78 | detected |
| 27 | II A | Recto-sigmoid junction | 70 | M | 39.32 | ND | ND | 31.84 | 31.87 | 32.14 | detected |
| 28 | II A | Recto-sigmoid junction | 70 | M | 37.86 | 37.94 | 37.59 | 31.46 | 31.62 | 31.50 | detected |
| 29 | II A | Rectum | 58 | M | 26.98 | 26.97 | 26.95 | 27.24 | 27.14 | 27.08 | detected |
| 30 | II A | Sigmoid | 69 | M | 33.71 | 33.72 | 32.81 | 31.44 | 31.25 | 31.24 | detected |
| 31 | II A | Cecum | 68 | M | ND | ND | ND | 31.51 | 31.61 | 31.44 | ND |
| 32 | II A | Transverse | 80 | M | 35.85 | 35.21 | 35.78 | 31.12 | 31.09 | 31.18 | detected |
| 33 | II A | Sigmoid | 75 | F | 39.14 | 37.27 | 37.39 | 31.69 | 31.79 | 31.75 | detected |
| 34 | II B | Sigmoid | 52 | M | ND | ND | ND | 30.84 | 30.80 | 31.01 | ND |
| 35 | II B | Recto-sigmoid junction | 73 | M | 37.83 | 39.47 | 36.86 | 31.19 | 31.22 | 31.23 | detected |
| 36 | II B | Rectum | 66 | F | 37.57 | 35.89 | 36.94 | 31.87 | 31.77 | 31.68 | detected |
| 37 | II B | Hepatic flexure | 59 | M | ND | ND | ND | 31.95 | 31.98 | 31.71 | ND |
| 38 | II B | Cecum | 55 | F | 39.29 | 39.52 | ND | 31.60 | 31.59 | 31.65 | detected |
| 39 | III | Not specified | 84 | F | ND | ND | 37.09 | 32.00 | 32.20 | 31.89 | detected |
| 40 | III | Not specified | 54 | F | 31.25 | 31.38 | 31.2 | 27.17 | 27.06 | 27.06 | detected |
| 41 | III | Rectum | 81 | M | 37.86 | 38.18 | 35.75 | 30.62 | 30.52 | 30.45 | detected |
| 42 | III | Rectum | 47 | F | 32.85 | 32.61 | 32.63 | 30.58 | 30.13 | 30.30 | detected |
| 43 | III | Cecum | 73 | F | 36.98 | 36.75 | 34.91 | 30.52 | 30.59 | 30.71 | detected |
| 44 | III | Rectum | 47 | M | 31.46 | 31.53 | 31.49 | 30.90 | 30.82 | 30.92 | detected |
| 45 | III | Rectum | 42 | F | 37.19 | 37.08 | 37.24 | 31.19 | 31.32 | 31.56 | detected |
| 46 | IV | Not specified | 49 | F | 34.32 | 34.31 | 35.21 | 31.36 | 31.57 | 31.68 | detected |
| 47 | IV | Recto-sigmoid junction | 57 | M | 31.34 | 31.50 | 31.27 | 30.08 | 30.08 | 29.92 | detected |
| 48 | IV | Sigmoid | 69 | F | 37.75 | 38.25 | 37.77 | 32.98 | 32.8 | 32.64 | detected |
| 49 | IV | Cecum | 53 | M | 32.25 | 32.14 | 32.03 | 30.00 | 29.84 | 29.87 | detected |
| 50 | IV | Splenic flexure | 63 | F | 37.86 | 37.41 | 36.30 | 30.62 | 30.50 | 30.55 | detected |

ND – not detected

CP – crossing point
