# Supplementary material for: Septin 9 methylated DNA is a sensitive and specific blood test for colorectal cancer
Source: BMC Med. 2011 Dec 14;9:133. doi: 10.1186/1741-7015-9-133 (PMC3271041; doi:10.1186/1741-7015-9-133)
Supplement: Additional file 3 — Supplementary Table 3. Control Plasma Specimens. [file 1741-7015-9-133-S3.DOC]

**Supplementary Table 3. Control Plasma Specimens**

| **ID** | **Age** | **Sex** | **Colonoscopy**  **Results** | ***SEPT9* CP 1** | ***SEPT9* CP 2** | ***SEPT9* CP 3** | ***ACTB***  **CP 1** | ***ACTB***  **CP 2** | ***ACTB***  **CP 3** | ***SEPT9* Detection** |
| --- | --- | --- | --- | --- | --- | --- | --- | --- | --- | --- |
| 1 | 66 | M | normal | ND | ND | ND | 32.16 | 32.24 | 32.23 | ND |
| 2 | 46 | M | normal | ND | ND | ND | 32.50 | 32.54 | 32.47 | ND |
| 3 | 41 | F | normal | ND | ND | ND | 31.21 | 31.66 | 31.71 | ND |
| 4 | 51 | F | normal | ND | 38.4 | ND | 30.79 | 30.87 | 31.07 | detected |
| 5 | 65 | F | normal | ND | ND | ND | 31.59 | 31.89 | 31.84 | ND |
| 6 | 51 | F | normal | ND | ND | ND | 30.75 | 30.94 | 30.85 | ND |
| 7 | 52 | F | normal | ND | ND | ND | 31.82 | 32.04 | 32.16 | ND |
| 8 | 48 | F | normal | ND | ND | ND | 32.34 | 32.09 | 32.44 | ND |
| 9 | 70 | M | normal | ND | ND | ND | 32.90 | 32.88 | 32.74 | ND |
| 10 | 60 | M | normal | ND | ND | ND | 32.18 | 31.96 | 31.96 | ND |
| 11 | 76 | M | normal | ND | ND | ND | 33.00 | 32.92 | 32.80 | ND |
| 12 | 70 | F | normal | ND | ND | ND | 31.61 | 31.49 | 31.06 | ND |
| 13 | 59 | M | normal | ND | ND | ND | 32.45 | 32.45 | 32.49 | ND |
| 14 | 51 | M | normal | ND | ND | ND | 32.44 | 32.46 | 32.42 | ND |
| 15 | 52 | F | normal | ND | ND | ND | 32.66 | 32.69 | 32.79 | ND |
| 16 | 52 | F | normal | ND | ND | ND | 34.65 | 34.63 | 34.70 | ND |
| 17 | 51 | M | normal | ND | ND | ND | 31.71 | 31.78 | 30.96 | ND |
| 18 | 51 | F | normal | ND | ND | ND | 30.83 | 30.82 | 30.79 | ND |
| 19 | 52 | F | normal | ND | ND | ND | 31.69 | 31.75 | 31.79 | ND |
| 20 | 50 | M | normal | ND | ND | ND | 31.06 | 31.14 | 31.42 | ND |
| 21 | 77 | F | normal | ND | ND | ND | 30.87 | 30.90 | 31.14 | ND |
| 22 | 61 | M | normal | ND | ND | ND | 31.57 | 31.48 | 31.69 | ND |
| 23 | 69 | M | normal | ND | ND | ND | 32.44 | 32.17 | 32.11 | ND |
| 24 | 51 | M | normal | ND | ND | ND | 31.58 | 31.65 | 31.66 | ND |
| 25 | 47 | F | normal | ND | ND | ND | 32.86 | 33.00 | 32.87 | ND |
| 26 | 62 | M | normal | ND | ND | ND | 31.08 | 30.92 | 31.17 | ND |
| 27 | 44 | M | normal | ND | ND | ND | 32.08 | 32.13 | 31.92 | ND |
| 28 | 69 | F | normal | ND | ND | ND | 31.52 | 31.46 | 31.65 | ND |
| 29 | 57 | M | normal | ND | ND | ND | 32.14 | 32.02 | 32.07 | ND |
| 30 | 53 | M | normal | ND | ND | ND | 32.42 | 32.12 | 32.17 | ND |
| 31 | 50 | F | normal | ND | ND | ND | 31.34 | 31.70 | 31.66 | ND |
| 32 | 53 | M | normal | ND | ND | ND | 31.65 | 31.48 | 31.7 | ND |
| 33 | 59 | F | normal | ND | ND | ND | 32.17 | 31.94 | 32.13 | ND |
| 34 | 51 | M | normal | ND | ND | ND | 31.48 | 31.60 | 31.70 | ND |
| 35 | 79 | M | normal | ND | ND | ND | 30.87 | 30.91 | 30.91 | ND |
| 36 | 78 | F | normal | 38.78 | ND | ND | 31.30 | 31.54 | 31.49 | detected |
| 37 | 54 | F | normal | ND | ND | ND | 30.17 | 30.18 | 30.10 | ND |
| 38 | 60 | F | normal | 44.49 | ND | ND | 30.61 | 30.46 | 30.32 | detected |
| 39 | 67 | F | normal | ND | ND | ND | 30.71 | 30.53 | 30.70 | ND |
| 40 | 64 | F | normal | ND | ND | ND | 30.23 | 30.17 | 30.26 | ND |
| 41 | 86 | M | normal | ND | ND | ND | 30.91 | 31.43 | 31.18 | ND |
| 42 | 61 | F | normal | ND | ND | ND | 32.05 | 32.45 | 32.62 | ND |
| 43 | 60 | M | normal | ND | ND | ND | 31.50 | 31.53 | 31.32 | ND |
| 44 | 51 | F | normal | ND | ND | ND | 31.77 | 31.73 | 31.95 | ND |
| 45 | 57 | F | normal | ND | ND | ND | 30.58 | 30.57 | 30.48 | ND |
| 46 | 54 | M | normal | ND | 38.66 | ND | 30.92 | 30.88 | 31.03 | detected |
| 47 | 57 | F | normal | ND | ND | ND | 32.26 | 32.45 | 32.35 | ND |
| 48 | 50 | F | normal | ND | ND | ND | 31.69 | 31.59 | 31.87 | ND |
| 49 | 83 | M | normal | ND | 37.74 | ND | 32.02 | 32.34 | 32.51 | detected |
| 50 | 61 | F | normal | ND | ND | ND | 32.48 | 32.68 | 32.53 | ND |
| 51 | 49 | M | normal | ND | ND | ND | 32.64 | 32.76 | 32.80 | ND |
| 52 | 44 | M | normal | ND | ND | ND | 31.88 | 31.91 | 31.81 | ND |
| 53 | 50 | M | normal | 37.16 | ND | ND | 31.77 | 31.81 | 31.72 | detected |
| 54 | 61 | M | normal | ND | ND | ND | 32.73 | 32.77 | 32.84 | ND |
| 55 | 40 | F | normal | ND | ND | ND | 32.20 | 32.16 | 32.15 | ND |
| 56 | 51 | F | normal | ND | ND | ND | 31.09 | 31.14 | 31.24 | ND |
| 57 | 58 | F | normal | ND | ND | 36.9 | 30.43 | 30.31 | 30.50 | detected |
| 58 | 56 | M | normal | ND | ND | ND | 32.14 | 32.35 | 32.46 | ND |
| 59 | 52 | F | normal | 38.35 | 38.29 | ND | 32.51 | 32.61 | 32.60 | detected |
| 60 | 67 | F | normal | ND | ND | ND | 33.64 | 33.86 | 33.99 | ND |
| 61 | 65 | F | normal | ND | ND | ND | 31.63 | 31.83 | 31.70 | ND |
| 62 | 54 | F | normal | ND | ND | ND | 31.10 | 31.23 | 31.25 | ND |
| 63 | 51 | F | normal | ND | ND | ND | 31.85 | 31.80 | 31.79 | ND |
| 64 | 50 | M | normal | ND | ND | ND | 29.44 | 29.42 | 29.59 | ND |
| 65 | 59 | M | normal | ND | ND | ND | 32.70 | 32.69 | 32.72 | ND |
| 66 | 70 | F | normal | ND | ND | ND | 31.66 | 31.81 | 31.66 | ND |
| 67 | 64 | F | normal | ND | ND | ND | 31.68 | 31.76 | 31.81 | ND |
| 68 | 50 | M | normal | ND | ND | ND | 32.71 | 32.45 | 32.79 | ND |
| 69 | 66 | M | normal | ND | ND | ND | 32.03 | 31.95 | 31.74 | ND |
| 70 | 65 | M | normal | ND | ND | ND | 31.28 | 31.30 | 31.45 | ND |
| 71 | 51 | F | normal | ND | ND | ND | 32.79 | 32.93 | 33.11 | ND |
| 72 | 57 | F | normal | ND | ND | ND | 31.09 | 31.13 | 30.99 | ND |
| 73 | 61 | M | normal | ND | ND | ND | 31.12 | 31.12 | 31.26 | ND |
| 74 | 41 | M | normal | ND | ND | ND | 31.09 | 31.03 | 31.08 | ND |
| 75 | 58 | F | normal | ND | ND | ND | 31.44 | 31.26 | 31.24 | ND |
| 76 | 60 | F | normal | ND | ND | ND | 31.18 | 31.18 | 31.19 | ND |
| 77 | 51 | M | normal | ND | ND | ND | 31.55 | 31.65 | 31.54 | ND |
| 78 | 61 | F | normal | ND | ND | ND | 31.60 | 31.73 | 31.80 | ND |
| 79 | 50 | F | normal | ND | ND | ND | 32.88 | 33.02 | 33.23 | ND |
| 80 | 60 | M | normal | ND | ND | ND | 31.28 | 31.19 | 31.21 | ND |
| 81 | 79 | F | normal | ND | ND | ND | 30.56 | 30.47 | 30.55 | ND |
| 82 | 72 | M | normal | ND | ND | ND | 31.96 | 31.89 | 32.20 | ND |
| 83 | 53 | F | normal | ND | ND | ND | 31.44 | 31.35 | 31.46 | ND |
| 84 | 52 | M | normal | ND | ND | ND | 31.48 | 31.27 | 31.34 | ND |
| 85 | 60 | F | normal | ND | 40.15 | ND | 31.55 | 31.66 | 31.18 | detected |
| 86 | 56 | F | normal | ND | ND | ND | 32.29 | 32.29 | 31.97 | ND |
| 87 | 61 | M | normal | ND | ND | ND | 31.73 | 31.60 | 31.68 | ND |
| 88 | 56 | F | normal | 39.76 | ND | ND | 30.68 | 30.88 | 31.04 | detected |
| 89 | 65 | F | normal | ND | ND | ND | 32.70 | 32.82 | 32.87 | ND |
| 90 | 58 | F | normal | ND | ND | ND | 31.98 | 31.98 | 32.07 | ND |
| 91 | 61 | M | normal | 39.28 | ND | ND | 31.86 | 31.70 | 31.95 | detected |
| 92 | 53 | F | normal | ND | ND | ND | 31.90 | 31.71 | 31.89 | ND |
| 93 | 65 | F | normal | ND | ND | ND | 30.84 | 31.14 | 30.92 | ND |
| 94 | 56 | M | normal | ND | ND | ND | 31.56 | 31.76 | 31.80 | ND |

ND – not detected

CP – crossing point
